# Rapid restitution of contractile dysfunction by synthetic copolymers in dystrophin-deficient single live skeletal muscle fibers

**DOI:** 10.1186/s13395-023-00318-y

**Published:** 2023-05-19

**Authors:** Dongwoo Hahn, Joseph D. Quick, Brian R. Thompson, Adelyn Crabtree, Benjamin J. Hackel, Frank S. Bates, Joseph M. Metzger

**Affiliations:** 1 Department of Integrative Biology & Physiology, Medical School, University of Minnesota, 6-125 Jackson Hall, 321 Church Street SE, Minneapolis, MN 55455 USA; 2 Chemical Engineering & Materials Science, University of Minnesota, 151 Amundson Hall, 421 Washington Avenue SE, Minneapolis, MN 55455 USA

**Keywords:** Duchenne muscular dystrophy, Dystrophin, Skeletal muscle, Membrane stabilizing copolymer, Contractile function, Calcium handling

## Abstract

Duchenne muscular dystrophy (DMD) is caused by the lack of dystrophin, a cytoskeletal protein essential for the preservation of the structural integrity of the muscle cell membrane. DMD patients develop severe skeletal muscle weakness, degeneration, and early death. We tested here amphiphilic synthetic membrane stabilizers in mdx skeletal muscle fibers (flexor digitorum brevis; FDB) to determine their effectiveness in restoring contractile function in dystrophin-deficient live skeletal muscle fibers. After isolating FDB fibers via enzymatic digestion and trituration from thirty-three adult male mice (9 C57BL10, 24 mdx), these were plated on a laminin-coated coverslip and treated with poloxamer 188 (P188; PEO_75_-PPO_30_-PEO_75_; 8400 g/mol), architecturally inverted triblock (PPO_15_-PEO_200_-PPO_15_, 10,700 g/mol), and diblock (PEO_75_-PPO_16_-C_4_, 4200 g/mol) copolymers. We assessed the twitch kinetics of sarcomere length (SL) and intracellular Ca^2+^ transient by Fura-2AM by field stimulation (25 V, 0.2 Hz, 25 °C). Twitch contraction peak SL shortening of mdx FDB fibers was markedly depressed to 30% of the dystrophin-replete control FDB fibers from C57BL10 (*P* < 0.001). Compared to vehicle-treated mdx FDB fibers, copolymer treatment robustly and rapidly restored the twitch peak SL shortening (all *P* < 0.05) by P188 (15 μM =  + 110%, 150 μM =  + 220%), diblock (15 μM =  + 50%, 150 μM =  + 50%), and inverted triblock copolymer (15 μM =  + 180%, 150 μM =  + 90%). Twitch peak Ca^2+^ transient from mdx FDB fibers was also depressed compared to C57BL10 FDB fibers (*P* < 0.001). P188 and inverted triblock copolymer treatment of mdx FDB fibers increased the twitch peak Ca^2+^ transient (*P* < 0.001). This study shows synthetic block copolymers with varied architectures can rapidly and highly effectively enhance contractile function in live dystrophin-deficient skeletal muscle fibers.

**Supplementary Information:**

The online version contains supplementary material available at 10.1186/s13395-023-00318-y.

## Introduction

Duchenne muscular dystrophy (DMD) is an X-linked progressive muscle-wasting disease caused by the lack of the cytoskeletal protein dystrophin [1]. Dystrophin maintains a mechanical tether between the cytoskeleton of muscle fibers and the extracellular matrix, functioning as an essential stabilizer of muscle fiber membrane [2]. The absence of this protein results in myofiber mechanical dysfunction and marked susceptibility to contraction-induced injury, oxidative stress, and muscle loss [3–6]. Membrane instability and abnormal permeability caused by dystrophin-deficiency triggers extracellular Ca^2+^ influx to the myofibers that initiates further deteriorative downstream pathways, including Ca^2+^-induced proteases, mitochondrial Ca^2+^ overload, and oxidative damage [7–10]. Thus, DMD displays progressive weakness in limb muscle and postural muscle [11] as well as respiratory muscle [12] causing exercise intolerance. DMD patients also suffer from dilated cardiomyopathy and arrhythmias, leading to fatal heart failure [13, 14].

Unfortunately, no cure or effective treatment is available to halt or reverse DMD disease progression. Current therapeutic strategies have shown some beneficial effects, including glucocorticoids [15], steroids [16], anti-inflammatory drugs in patients [17], and gene therapy in animal studies [18–20]. In the last several decades, some of these approaches, especially corticosteroids, have become standard care for DMD. However, these treatments also bear significant side effects such as weight gain, adverse behavioral changes, and osteoporosis [15]. Furthermore, the improvements by these approaches have shown high variability among DMD patients [21, 22]. As many therapeutic approaches are specific to skeletal muscle, the discordant treatments between skeletal and cardiac muscle might also trigger elevated stress and cardiomyopathy [23]. The pathophysiological defects of DMD originate from the fragile muscle membrane to mechanical stress. Hence, there is a great need for the development of alternative therapeutic approaches that specifically target the dystrophin-deficient muscle membrane, such as synthetic membrane stabilizing copolymers [24, 25].

Poloxamer 188 (P188) is a linear nonionic triblock copolymer formed by a hydrophobic block of poly(propylene oxide) (PPO) flanked by two hydrophilic blocks of poly(ethylene oxide) (PEO). Recently, there has been great interest to study and implement synthetic copolymers in DMD due to their protective effects on the muscle cell membrane. P188 is a first-in-class molecule and has shown its protective effects in neurons with excitotoxic and oxidative injury [26], in skeletal muscle fibers with electrical injury [27–29], mucosa with ischemia/reperfusion injury [30], erythrocytes membranes with radiation injury [31], fibroblasts with heat injury [32], and in sickle cell disease [33, 34]. These studies utilized the innate structure of the phospholipid bilayer of the cells and its interaction with amphiphilic copolymer [35]. Thus, the aforementioned fragility in the muscle cell membranes in DMD makes these patients prominent candidates for therapies featuring membrane interfacing copolymers. Indeed, an acute delivery of P188 to isolated cardiac myocytes from dystrophin-deficient mdx mice blocked aberrant Ca^2+^ influx and altered cell compliance in vitro [36]. In addition, this study demonstrated that in vivo administration of P188 to mdx mice improved the myocardial pressure–volume relationship as well as survival upon the dobutamine-induced stress. Another study found that subcutaneous delivery of P188 successfully decreased the membrane damage and lengthening contraction-induced force loss in the hindlimb muscles from mdx mice [24].

Whereas there have been promising findings in applying copolymers in the dystrophin-deficient cardiac and skeletal muscle, there is a gap in the literature regarding developing and implementing a facile skeletal muscle primary cell system for testing DMD therapeutics. Hence, the field will be advanced by the development and application of a single mature skeletal muscle fibers primary culture system for investigating mechanistic structure–function questions using copolymers. Furthermore, poloxamers can be manufactured in diverse forms by differing ratios of PEO:PPO blocks and molecular weight [37]. However, the structure–function relationship of these copolymers has not been fully addressed. In this study, we investigated diblock (PEO-PPO) and inverted triblock copolymer (PPO-PEO-PPO), which have distinct architectures compared to P188 (PEO-PPO-PEO), to test their effectiveness in the rescue of contractile functional deficits in the dystrophin-deficient skeletal muscle fibers. The comparison of the effectiveness of differing copolymer structures will shed new light on the mechanism regarding how the block copolymers stabilize the dystrophin-deficient skeletal muscle membranes.

The objectives of this study were to assess the phenotypic differences in the contractile function due to the absence of dystrophin and to investigate the protective effects of copolymers with diverse architectures in the restoration of contractile function from primary, live, dystrophin-deficient skeletal muscle fibers. Here, we applied P188, diblock, and inverted triblock PPO-PEO-PPO copolymers to dystrophin-deficient live isolated skeletal muscle fibers in a mouse model of DMD and tested effects on contractile function and intracellular Ca^2+^ kinetics. Results showed that the application of these three synthetic membrane-stabilizing copolymers rapidly improved the contractile functional deficits of the isolated dystrophin-deficient skeletal muscle fibers up to 90% of dystrophin-intact controls. Enhanced skeletal muscle fiber performance by P188 as well as the ability of P188 and inverted triblock copolymers to improve twitch peak intracellular Ca^2+ ^ transient suggest that there are both Ca^2+^-dependent and Ca^2+^-independent mechanisms in the copolymer-dependent restoration of the contractile function from the skeletal muscle fibers from mdx mice.

## Methods

### Animals

We used a total of 33 male C57BL/10ScSn-*DMD*^*mdx*^/J (mdx) and its genetic background C57BL/10ScSnJ (BL10) mice, all aged 6–12 months from Jackson Laboratory. The mdx strain lacks dystrophin protein and provides a well-studied model of Duchenne muscular dystrophy [38, 39]. Males were used as Duchenne muscular dystrophy is an X-linked disease. Two to three mice were assigned to each group, which consisted of 33–59 isolated fibers per group. We housed the mice at the University of Minnesota Animal Care Facilities in a 12:12-h light–dark cycle and supplied standard chow and water ad libitum. The protocol was approved by the Institutional Animal Care and Use Committee of the University of Minnesota.

### Isolation of flexor digitorum brevis

We harvested muscle samples from one animal per day at the same time of the day (8:30 AM CST). After euthanizing the mice with isoflurane overdose and cervical dislocation, we immediately dissected the flexor digitorum brevis (FDB) from both hind paws and enzymatically digested by incubation in M199 (M3769, Sigma-Aldrich; pH 7.4) supplemented with 0.2 g/L bovine serum albumin (A9647, Sigma-Aldrich) and 4.6 mM HEPES (BP310-1, Fisher Scientific) containing 0.2% collagenase type II (LS004176, Worthington Biochemical Corporation) and 10% fetal bovine serum at 37 °C for 3 h. Next, we separated single myofibers with trituration by using Pasteur pipettes and plated the fibers on 20 μg/ml laminin-coated coverslips for 1 h at 37 °C in 5% CO_2_. The myofibers were cultured in M199 (31,100–035, Life Technology; pH 7.4) supplemented with 26.1 mM sodium bicarbonate (S5761, Sigma-Aldrich), 22.9 mM HEPES (BP310-1, Fisher Scientific), 0.2 g/L bovine serum albumin (A9647, Sigma-Aldrich), 1000 μ/ml Penicillin–streptomycin (1514–122, Gibco), ITS media supplement (I1884, Sigma-Aldrich; 5 mg/L insulin, 5 mg/L transferrin, 5 μg/L sodium selenite) for 30 min. Isolated myofibers were tested within 3 h after sample preparation. The cellular morphometrics of live isolated myofibers on laminin-coated coverslips was measured from microscopic images acquired with × 20 objective (BZ-X810, Keyence) by using ImageJ (NIH).

### Microscopic imaging of isolated live single FDB fibers

We isolated FDBs as described above and fixed the slides with 4% paraformaldehyde for 20 min at room temperature. After washing with PBS 2 × 5 min, we blocked the samples with PBS containing 5% bovine serum albumin (A9647, Sigma-Aldrich) and 0.5% Triton X-100 for 1 h. We probed the coverslips with a primary antibody for α-actinin (32,462, 1:500, Novus) for 1 h at room temperature. After washing with PBS 3 × 5 min, we incubated the coverslips with secondary antibody Goat Anti-Rabbit 647 (A-21244, 1:500, ThermoFisher) for 1 h. After washing with PBS 3 × 5 min, we applied ProLong™ Diamond Antifade Mountant (P36965, Invitrogen) to attach coverslips to the glass slide. Slides were allowed to cure for 1 day at room temperature in the dark. We used a fluorescence microscope (BZ-X810, Keyence) and a confocal microscope with 640 nm laser (C2, Nikon) in the University Imaging Center of the University of Minnesota.

### Copolymers synthesis and characterization

The molecular characteristics of three copolymers with different engineered structures are specified in Table 1. Compared to the P188, the diblock and inverted triblock copolymers have different molecular weights but very similar PEO weight ratios. The most distinct feature of these copolymers is the relative location of PEO and PPO as illustrated by the structures in Table 1. The details of molecular characterization of the diblock and inverted triblock copolymers can be found in the supplemental information (Figure S1).

**Table 1:** Membrane stabilizing block copolymers with diverse molecular structures

Copolymer	Chemical formula	Molecular weight	PEO weight %	Structure
P188	PEO_75_-PPO_30_-PEO_75_	8,400 g/mol	80%	
Diblock	PEO_75_-PPO_16_-C(CH_3_)_3_	4,200 g/mol	77%	
Inverted	PPO_15_-PEO_200_-PPO_15_	10,700 g/mol	83%	

Poly(ethylene oxide): PEO, hydrophilic

Poly(propylene oxide): PPO, hydrophobic

#### P188

Poloxamer 188 (P188; PEO_75_-PPO_30_-PEO_75_, 8400 g/mol, 80% PEO weight %) in National Formulary grade was provided by BASF.

#### Diblock copolymer

We used high purity (> 99.0%) monomers (propylene oxide and ethylene oxide), potassium tert-butoxide, 18-crown-6 ether (Sigma), and dihydroxy terminated PEO (8000 g/mol) (Polymer Source) for the synthesis of the diblock copolymer.

Anionic polymerization was employed to create the diblock copolymer (diblock; PEO_75_-PPO_16_-C(CH_3_)_3_, 4200 g/mol, 77% PEO weight %). Details about the polymer synthesis are described elsewhere [40]. Reactions were carried out in an inert, argon atmosphere with alumina column-dried tetrahydrofuran (THF) as the solvent. At room temperature, propylene oxide was initiated by potassium t-butoxide and allowed to react in the presence of 18-crown-6 ether at a 2:1 molar ratio to the initiator. The inclusion of 18-crown-6 ether reduces products from unwanted side reactions and increases conversion [41]. After 120 h, the reaction was terminated by the addition of acidic methanol (10:1 methanol: 37 w/w% hydrochloric acid). Removal of potassium and 18-crown-6 ether was accomplished by repeated filtering, evaporation, and dissolution in THF followed by a hexane-water extraction. The tertiary butyl end-functionalized PPO (t-PPO) was re-initiated with potassium naphthalenide. Ethylene oxide was added and allowed to react for 48 h and terminated similarly. Repetitive filtering, evaporation, and dissolution in THF removed any remaining potassium and impurities from the polymer.

#### Inverted triblock copolymer

Inverted triblock copolymer (PPO_15_-PEO_200_-PPO_15_, 10,700 g/mol, 83% PEO weight %) was made similarly by initiating dihydroxy terminated PEO with potassium naphthalenide followed by addition of propylene oxide monomer and reaction at room temperature for 120 h in the presence of 18-crown-6 ether. Termination was effected with acidic methanol. Repetitive filtering, evaporation, and dissolution in tetrahydrofuran followed by the use of desalting columns removed impurities.

We prepared 30 μM and 300 μM solutions with the above three copolymers in deionized water. On the day of the experiment, these copolymer solutions were mixed with supplemented cell culture medium M199 at × 2 concentration in a 1:1 volume ratio, to make final concentrations of 15 μM and 150 μM. Finally, we incubated the isolated FDB fibers with 15 μM and 150 μM copolymer solutions in M199 at 25 °C for 10 min. Untreated myofibers from BL10 and mdx mice were incubated with the same batch of M199 in × 2 concentration mixed with deionized water in a 1:1 volume ratio as a vehicle.

### Sarcomere length kinetics and Ca^2+^ handling measurement

We used the IonOptix Calcium and Contractility System connected with MyoCam-S (IonOptix) in an inverted microscope with a 40× objective to measure sarcomere length kinetics and fluorescence photometry. IonOptix calculates the average frequency of the sinusoidal appearance by altering striation patterns of dark (A-band) and light (I-band) from isolated single myofibers. We applied 250 Hz sampling frequency for sarcomere length kinetics and 1000 Hz for fluorescence photometry with 25 V, 8 ms pulse duration (supplemental information Figure S3), 0.2 Hz field stimulation frequency at 25 °C. This stimulation achieved peak twitch contractions in isolated FDBs which is known to be sensitive to different electrical field configurations and pulse parameters [42]. For myoplasmic Ca^2+^ transients, the isolated FDB fibers were loaded with 1 μM Fura-2AM (F1221, Invitrogen) in M199 for 10 min at room temperature, and 15 min in M199 for deesterification in the dark. Sarcomere length kinetics and Fura-2AM signals were measured simultaneously. Our data with Fura-2 as an intracellular Ca^2+^ indicator compares well with a recent report using Fura-2 in skeletal muscle [43].

### Statistics

We performed the Shapiro–Wilk normality test and Brown-Forsythe equal variance test. If the data passed the normality test and equal variance test, we then compared groups by using Student’s *t* test. If the data failed the equal variance test, we applied log transformation. To compare among groups, we performed one-way ANOVA test with Holm-Sidak pairwise multiple post hoc comparisons or one-way ANOVA rank test (SigmaPlot ver. 14, Systat Software). In all cases, we used two-tailed tests with significant difference set at *P* < 0.05. Data were displayed as mean ± SD and figures in scatterplots (Prism ver. 6, GraphPad).

## Results

### Microscopic analysis of isolated FDB fibers

**Fig. 1:**
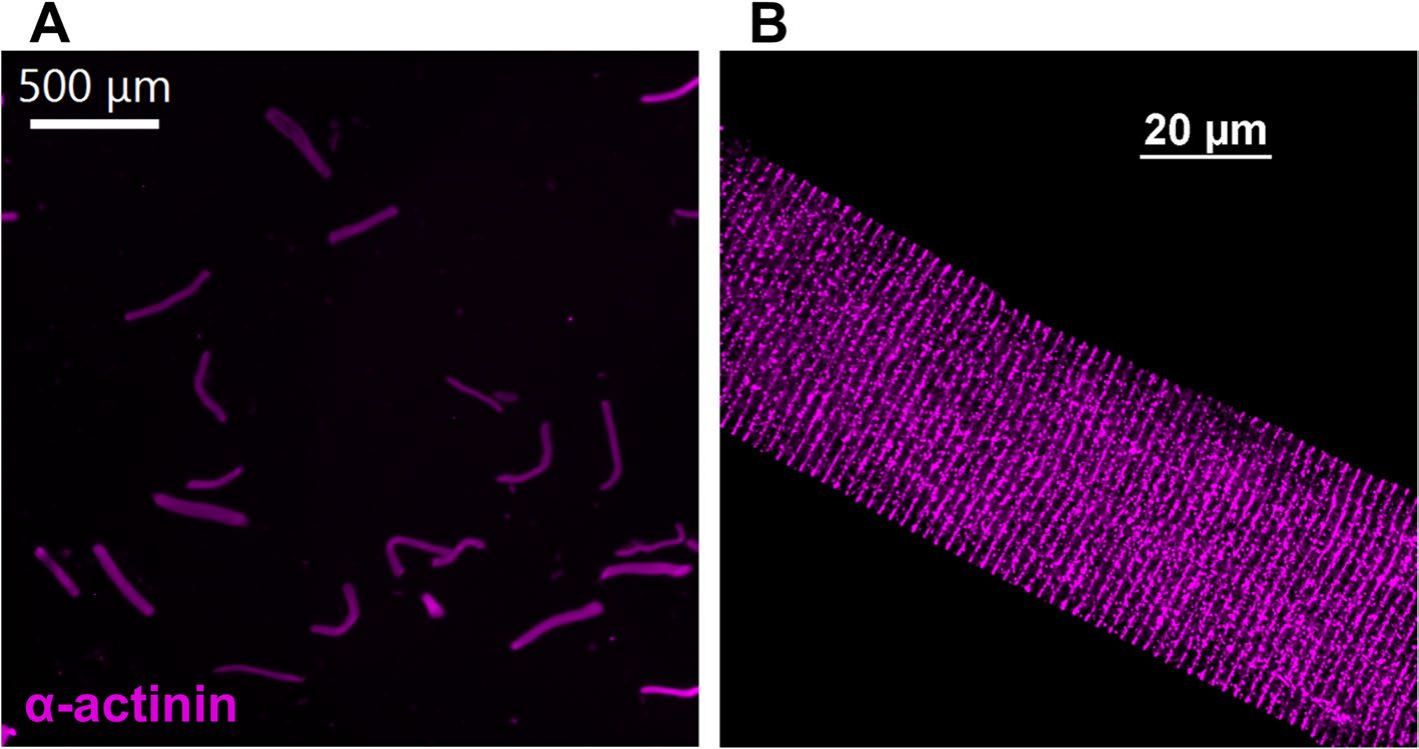
Immunofluorescence images of mouse flexor digitorum brevis (FDB) fibers (purple = α-actinin). **A** Isolated FDB fibers at low magnification. (scale bar = 500 μm). **B** Immunofluorescence image of isolated FDB fibers at high magnification by confocal microscopy (scale bar = 20 μm)

We isolated primary, live, membrane-intact flexor digitorum brevis (FDB) muscle fibers by enzymatic digestion and trituration for this study. Figure 1 shows the microscopic view of FDB fibers and displays the morphology of the isolated single FDB fibers with preserved striation pattern by α-actinin. The dimensions of live isolated FDB fiber from male C57BL10 (*n* = 45) and mdx (*n* = 61) were as follows (mean ± SD): diameter (C57BL10 = 33 ± 10 μm, mdx = 34 ± 11 μm; *P* = 0.834), length (C57BL10 = 365 ± 103 μm, 414 ± 103 μm; *P* = 0.018), cross-sectional area (C57BL10 = 944 ± 651 μm^2^, mdx = 982 ± 628 μm^2^; *P* = 0.762).

### Sarcomere length shortening and Ca^2+^ handling kinetics: C57BL10 vs. mdx

**Fig. 2:**
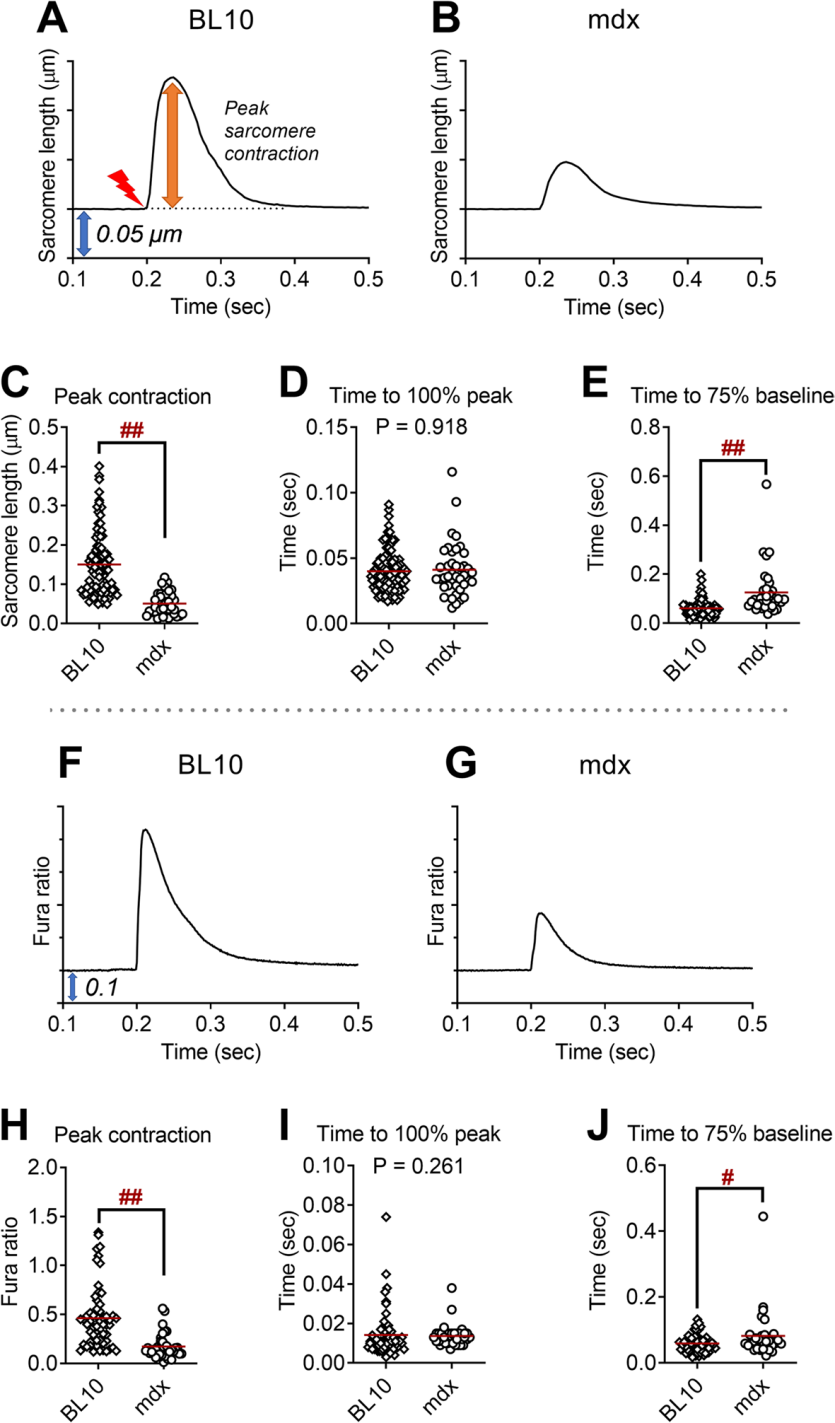
Twitch sarcomere length kinetics and Ca^2+^ transient signals from isolated, single dystrophin-intact flexor digitorum brevis (FDB) from C57BL10 (BL10; 6 mice, 93 fibers) and dystrophin-deficient FDB fibers from mdx mice (3 mice, 40 fibers). **A**, **B** Representative ensemble average of sarcomere length shortening patterns from FDB of BL10 and mdx mice. Sarcomere length on the *Y*-axis is inverted. Each tick on the *Y*-axis is 0.05 μm (blue arrow). Electric field stimulation was applied at 0.2-s point (red mark). **C** Peak sarcomere length shortening during twitch contraction. **D** Time to peak sarcomere length shortening during twitch contraction. **E** Time from peak to 75% of baseline of sarcomere length during twitch contraction. **F**, **G** Ensemble average of Ca^2+^ transient patterns measured by Fura-2AM fluorescence from FDB of BL10 and mdx mice. Each tick on the *Y*-axis is 0.1 (blue arrow). **H** Peak Ca^2+^ transient during twitch contraction. **I** Time to peak Ca^2+^ transient during twitch contraction. **J** Time from peak to 75% of baseline of Ca^2+^ transient during twitch contraction. ^#^*P* < 0.05, ^##^*P* < 0.001 by Student’s *t* test

Initially, we sought to quantify the phenotypic differences in the contractile function of the skeletal muscle fiber caused by the absence of dystrophin protein. Thus, we compared the sarcomere length shortening kinetics and Ca^2+^ handling kinetics in FDB fibers from adult male C57BL10 and mdx mice at 25 °C. There was no statistical difference in the baseline sarcomere length between control and mdx FDB fibers (C57BL10 = 1.946 ± 0.060 μm, mdx = 1.928 ± 0.063 μm; *P* > 0.05). In addition, baseline Ca^2+^ was not significantly different between control and mdx FDB fibers (360/380 ratios: C57BL10 = 1.107 ± 0.145, mdx = 1.044 ± 0.162; *P* > 0.05). As represented by average sarcomere shortening patterns in Fig. 2A,B, the peak twitch sarcomere length shortening of FDB fibers from mdx was 70% lower than in C57BL10 (*P* < 0.001, Fig. 2C). Furthermore, FDB fibers from mdx displayed a lower peak twitch Ca^2+^ transient compared to C57BL10 (*P* < 0.001, Fig. 2H). Interestingly, FDB fibers from mdx showed 40% slower Ca^2+^ reuptake kinetics represented by time to 75% baseline (*P* < 0.05, Fig. 2J). These findings illustrate that the lack of dystrophin protein induces severe dysfunction in contraction as well as Ca^2+^ handling deficits in the single FDB fibers from adult mdx mice.

### Sarcomere length shortening and Ca^2+^ handling kinetics: P188

**Fig. 3:**
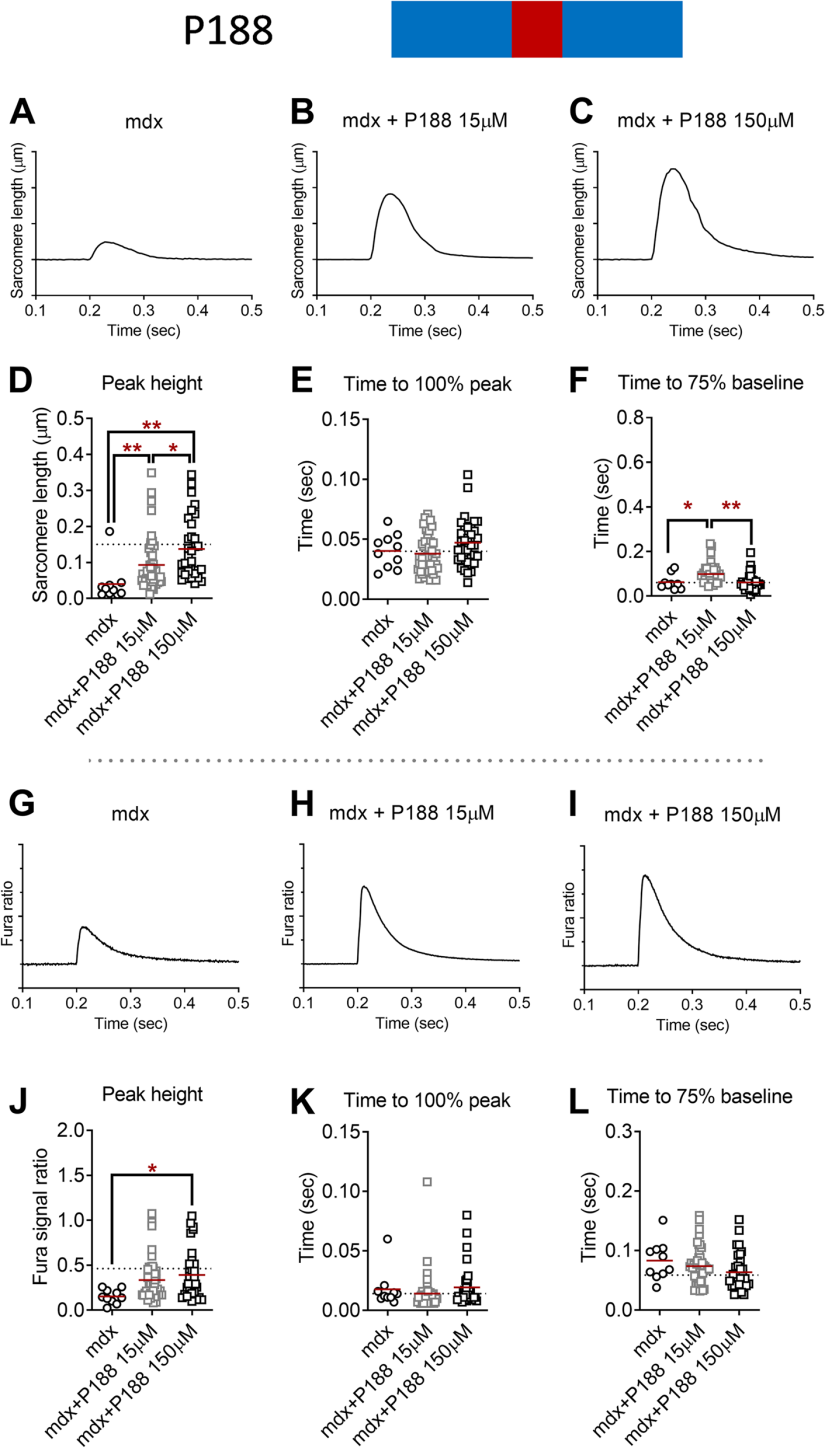
Twitch sarcomere length kinetics and Ca^2+^ transient signals of dystrophin-deficient flexor digitorum brevis (FDB) fibers from mdx mice treated with vehicle (1 mouse, 10 fibers) and P188 (3 mice each; 15 μM, 48 fibers; 150 μM, 33 fibers). **A**–**C** Representative ensemble average of sarcomere length shortening patterns from mdx FDB treated with vehicle and P188 (15 μM, 150 μM). Sarcomere length on the *Y*-axis is inverted. Each tick on the *Y*-axis is 0.05 μm. Electric field stimulation was applied at 0.2-s point. **D** Peak sarcomere length shortening during twitch contraction. **E** Time to peak sarcomere length shortening during twitch contraction. **F** Time from peak to 75% of baseline of sarcomere length during twitch contraction. **G**–**I** Ensemble average of Ca^2+^ transient patterns measured by Fura-2AM fluorescence from mdx FDB fibers treated with vehicle and P188 (15 μM, 150 μM). Each tick on the *Y*-axis is 0.1. **J** Peak Ca^2+^ transient during twitch contraction. **K** Time to peak Ca^2+^ transient during twitch contraction. **L** Time from peak to 75% of baseline of Ca^2+^ transient during twitch contraction. Dotted lines represent the average values from BL10 FDB (**D**–**F**, **J**–**L**). *P < 0.05, **P < 0.001 by one-way ANOVA with Holm-Sidak’s post hoc test

We applied the triblock copolymer P188 to the isolated FDB fibers from adult male mdx mice. Remarkably, P188 treatment elicited 2.1-fold for 15 μM P188 and 3.2-fold for 150 μM P188 increases in the twitch peak sarcomere length shortening compared to the vehicle-treated mdx FDB fibers (*P* < 0.001 both, Fig. 3D). This functional improvement was rapid, and as fast as we could measure in this system (~ 10 min). These are equivalent to 62% (15 μM P188) and 92% (150 μM P188) of dystrophin-intact FDB fibers from C57BL10 (dotted line in Fig. 3D). Interestingly, mdx FDB fibers treated with 15 μM P188 showed significantly slower sarcomere length relaxation kinetics compared to mdx FDB fibers treated with vehicle (*P* < 0.05, Fig. 3F) and 150 μM P188 (*P* < 0.001, Fig. 3F). We also found 2.2-fold for 15 μM P188 (*P* = 0.10) and 2.6-fold for 150 μM P188 (*P* < 0.05) increased twitch peak Ca^2+^ transients compared to the vehicle-treated mdx FDB fibers (Fig. 3J). In additional experiments, C57BL/10 control FDB fibers were isolated and tested in pairwise format before and after 150 μM P188. Data show no significant effect of P188 on control fiber contraction amplitude (Supplemental Figure S2), in contrast to effects shown in mdx fibers (Fig. 3D).

### Sarcomere length shortening and Ca^2+^ handling kinetics: diblock copolymer

**Fig. 4:**
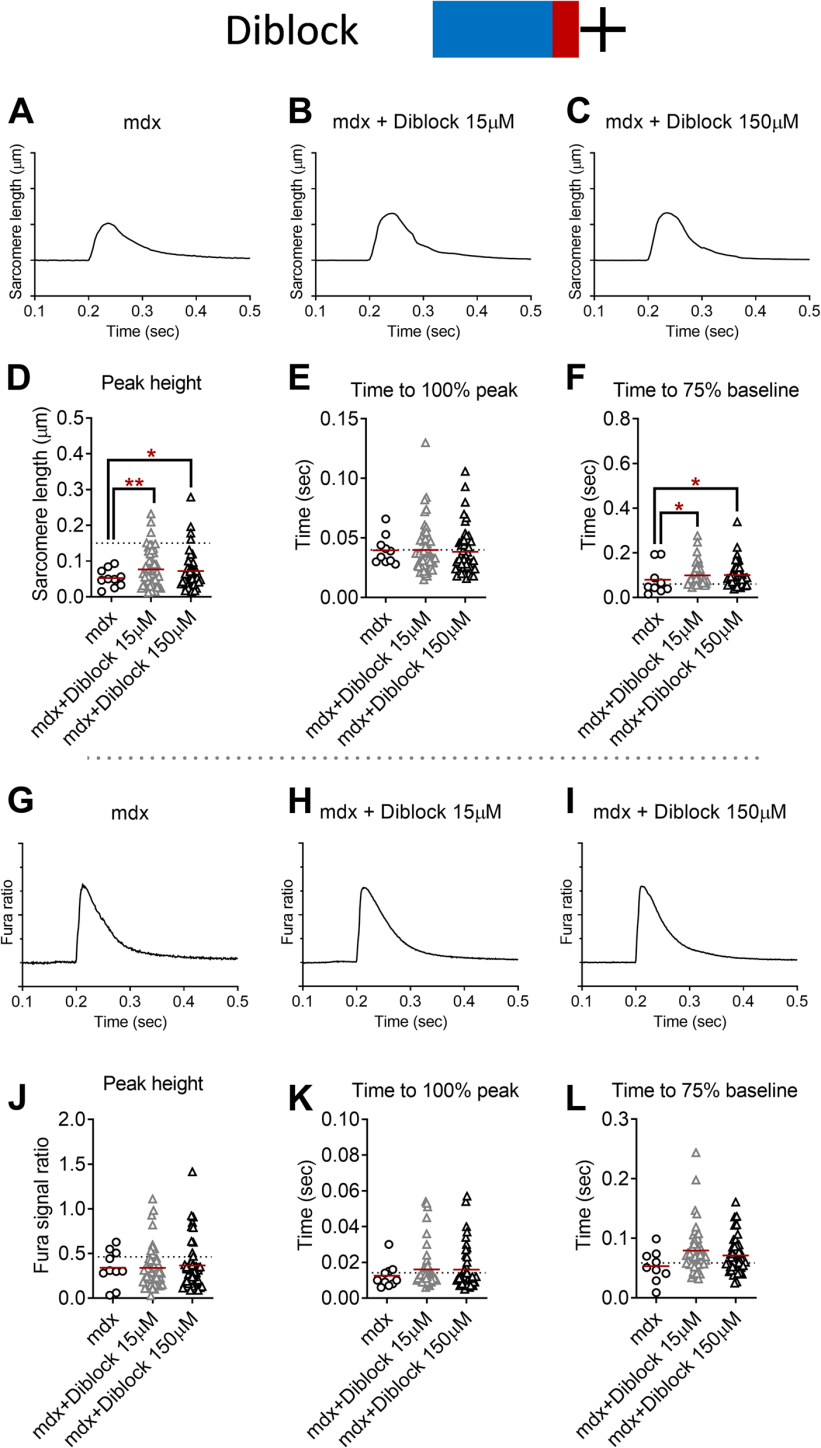
Twitch sarcomere length kinetics and Ca^2+^ transient signals of dystrophin-deficient flexor digitorum brevis (FDB) fibers from mdx mice treated with vehicle (1 mouse, 10 fibers) and diblock copolymer (3 mice each; 15 μM, 53 fibers; 150 μM, 45 fibers). **A**–**C** Representative ensemble average of sarcomere length shortening patterns from mdx FDB treated with vehicle and diblock copolymer (15 μM, 150 μM). Sarcomere length on the *Y*-axis is inverted. Each tick on *Y*-axis is 0.05 μm. Electric field stimulation was applied at 0.2-s point. **D** Peak sarcomere length shortening during twitch contraction. **E** Time to peak sarcomere length shortening during twitch contraction. **F** Time from peak to 75% of baseline of sarcomere length during twitch contraction. **G**–**I** Ensemble average of Ca^2+^ transient patterns measured by Fura-2AM fluorescence from mdx FDB fibers treated with vehicle and diblock copolymer (15 μM, 150 μM). Each tick on the *Y*-axis is 0.1. **J** Peak Ca^2+^ transient during twitch contraction. **K** Time to peak Ca^2+^ transient during twitch contraction. **L** Time from peak to 75% of baseline of Ca^2+^ transient during twitch contraction. Dotted lines represent the average values from BL10 FDB (**D**–**F**, **J**–**L**). *P < 0.05, **P < 0.001 by one-way ANOVA with Holm-Sidak’s post hoc test

In parallel to the P188 studies, we applied in separate experiments the diblock copolymer to isolated FDB fibers from adult male mdx mice. As displayed in Fig. 4D, we detected 50% greater twitch peak sarcomere length shortening with diblock copolymer at 15 μM (*P* < 0.001) and 150 μM (*P* < 0.05) compared to vehicle-treated mdx FDB fibers, respectively. These values were approximately 50% of dystrophin-intact FDB fibers from C57BL10 (dotted line in Fig. 4D). We also found 25% slower sarcomere length relaxation kinetics in mdx FDB fibers treated with diblock in both 15 μM and 150 μM compared to the vehicle-treated mdx fibers (*P* < 0.05 both, Fig. 4F). In contrast to P188, there were no statistical differences in the peak twitch Ca^2+^ transient signals or Ca^2+^ handling kinetics by diblock copolymer treatment (Fig. 4J–L).

### Sarcomere length shortening and Ca^2+^ handling kinetics: inverted triblock copolymer

**Fig. 5:**
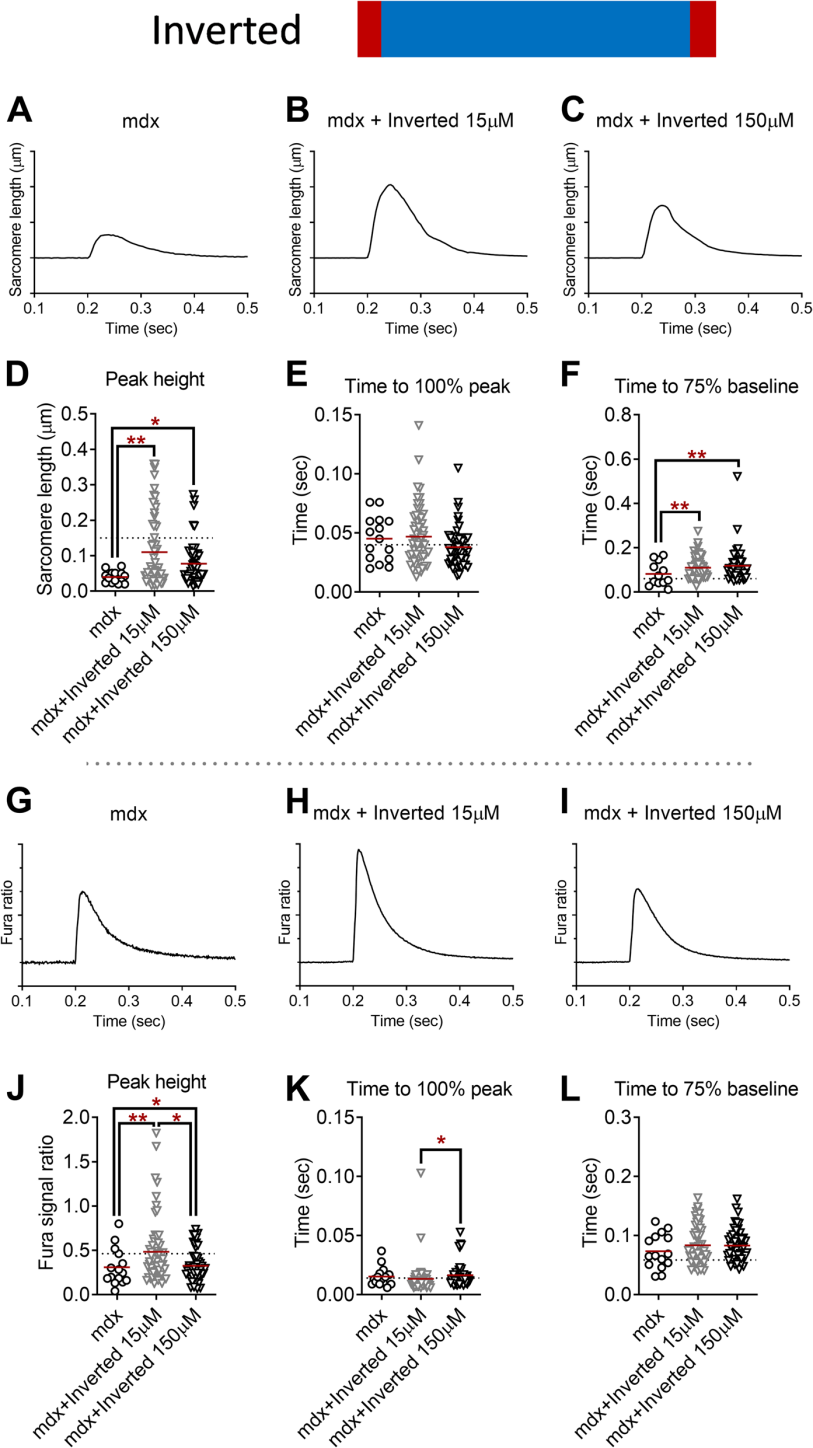
Twitch sarcomere length kinetics and Ca^2+^ transient signals of dystrophin-deficient flexor digitorum brevis (FDB) fibers from mdx mice treated with vehicle (1 mouse, 15 fibers) and inverted copolymer (3 mice each; 15 μM, 60 fibers; 150 μM, 52 fibers). **A**–**C** Representative ensemble average of sarcomere length shortening patterns from mdx FDB treated with vehicle and inverted copolymer (15 μM, 150 μM). Sarcomere length on the *Y*-axis is inverted. Each tick on *Y*-axis is 0.05 μm. Electric field stimulation was applied at 0.2-s point. **D** Peak sarcomere length shortening during twitch contraction. **E** Time to peak sarcomere length shortening during twitch contraction. **F** Time from peak to 75% of baseline of sarcomere length during twitch contraction. **G**–**I** Ensemble average of Ca^2+^ transient patterns measured by Fura-2AM fluorescence from mdx FDB fibers treated with vehicle and inverted copolymer (15 μM, 150 μM). Each tick on the *Y*-axis is 0.1. **J** Peak Ca^2+^ transient during twitch contraction. **K** Time to peak Ca^2+^ transient during twitch contraction. **L** Time from peak to 75% of baseline of Ca^2+^ transient during twitch contraction. Dotted lines represent the average values from BL10 FDB (**D**–**F**, **J**–**L**). *P < 0.05, **P < 0.001 by one-way ANOVA with Holm-Sidak’s post hoc test

Lastly, we treated isolated FDB fibers from adult male mdx mice with an inverted triblock copolymer. Similar to the above P188 and diblock copolymer studies, there was a 2.8-fold, for 15 μM inverted triblock copolymer (*P* < 0.001), and 1.9-fold, for 150 μM inverted copolymer (*P* < 0.05), increase in twitch peak sarcomere length shortening compared to vehicle-treated mdx FDB fibers (Fig. 5D). These data were 73% and 52% of the dystrophin-intact FDB fibers from C57BL10 (dotted line in Fig. 5D). The inverted triblock copolymer also prolonged the sarcomere length relaxation kinetics to 30–50% (*P* < 0.001 both, Fig. 5F). Peak twitch Ca^2+^ transient was increased by 60% for 15 μM inverted triblock copolymer (*P* < 0.001) and 6% for 150 μM inverted copolymer (*P* < 0.05) compared to vehicle-treated mdx FDB fibers (Fig. 5J). Inverted triblock copolymer at 150 μM showed 23% increased time to twitch peak Ca^2+^ transient compared to 15 μM (*P* < 0.05, Fig. 5K).

## Discussion

The main findings of this study are that synthetic membrane stabilizing copolymers, including triblock P188, diblock, and inverted triblock copolymers, rapidly improve the contractile function of the isolated dystrophin-deficient live skeletal muscle fibers. Remarkably, these effects of copolymers represent over a threefold improvement in the twitch sarcomere length shortening in the FDB fibers from adult male mdx mice, and this reached 90% of the dystrophin-replete FDB fibers from C57BL10 mice. Enhanced contractile performance was essentially instantaneous with copolymer treatment (as fast as could be measured in this system, < 10 min). In line with the sarcomere shortening, synthetic membrane copolymers increased the twitch peak Ca^2+^ transient in the mdx FDB fibers. The improvement of contractile function by copolymers showed differential effects on the Ca^2+^ transient amplitude, as discussed below. Overall, this study demonstrates that synthetic membrane stabilizing copolymers rescue twitch contractile dysfunction in dystrophin-deficient skeletal muscle fibers. This is the first study, to our knowledge, demonstrating the rapid restitution of the contractile function in the primary, live, membrane-intact dystrophin-deficient skeletal muscle.

### Rapid restitution in contractile dysfunction by copolymers

The application of synthetic amphiphilic copolymers to treat the fragile muscle membrane in DMD has been demonstrated in numerous studies [24, 26–28, 32, 36, 37, 44–49]. As a representative copolymer in this field, P188 is a nonionic linear triblock copolymer structured as a hydrophobic PPO core flanked by hydrophilic PEO tails on each end. This amphiphilic feature of the P188 protects the fragile membrane of the muscle fibers, thus functioning as a synthetic membrane stabilizer [50]. The protective effects of P188 have been reported in dystrophic heart and skeletal muscle and in cell culture models, in both acute and chronic application [24, 36, 37]. Similarly, copolymers with different architectures have been tested. For example, diblock copolymer (PEO_75_-PPO_15_) with modified chemical end groups (-C_4_, -H) were applied to C2C12 myoblasts with a hypoosmotic stress test as well as in mdx skeletal muscle via lengthening contractions in vivo [25, 51]. These studies report a significant protective effect of diblocks with the -C_4_ end group, indicating relative hydrophobicity, as modified by PPO end group, significantly influences the effectiveness of membrane stabilization. Another study applied higher molecular weight analog of  P188 (PEO_140_-PPO_44_-PEO_140_, 14,600 g/mol) and reported partial protection against hypoosmotic stress and lengthening contraction-induced force loss [24].

In this study, we applied membrane stabilizing copolymers in primary, live, membrane-intact single skeletal muscle fibers. All three synthetic copolymers, representing distinct architectures, including P188, diblock copolymer, and inverted triblock copolymer, showed 1.5- to 3.2-fold improvements in the twitch sarcomere length shortening from mdx FDB fibers. Importantly, these values attained over 90% of the twitch sarcomere length shortening in dystrophin-intact FDB fibers from healthy mice. Another important aspect of our findings is that this improvement was achieved rapidly, essentially immediately upon exchanging media with membrane stabilizing copolymers and re-testing contractile function (< 10 min). This rapid effect is further evidence of a physical interaction between copolymer and the phospholipid membrane underlying enhanced function, and not requiring new gene transcription/new protein translation.

### Potential mechanism of copolymers in sarcomere length shortening and Ca^2+^ handling restitution

As a potential mechanism underlying synthetic membrane stabilizing copolymers in the rapid restoration of sarcomere length shortening, the data suggest two mechanisms that are not mutually exclusive: Ca^2+^-dependent and Ca^2+^-independent. As discussed above, abnormal Ca^2+^ handling is one of the key pathophysiological components in dystrophin-deficient muscle fibers. Ca^2+^ handling dysfunction in mdx FDB fibers could be attributed to the abnormal excitability by the unstable muscle membrane. As the action potential regulates the excitation–contraction coupling process, its modification inevitably would affect the consequent muscle contractile function [52]. In line with this, the muscle membrane of DMD patients shows alterations in the internal molecular architecture and an ineffective cellular barrier [53, 54]. Neuromuscular transmission failure and reduced muscle excitability were also found to result in decreased force generation in the skeletal muscle from mdx mice [55–57]. Impairment in Ca^2+^ release from the sarcoplasmic reticulum was also observed in the isolated extensor digitorum longus (EDL) and FDB from mdx mice [58]. Our data also showed that untreated FDB fibers from mdx mice have a lower peak Ca^2+^ transient compared to BL10, and this may be explained by the reduced action potential and abnormal Ca^2+^ release from sarcoplasmic reticulum reported by others in the mdx myofibers [59].

We report a significant increase in the twitch peak Ca^2+^ transient in the mdx FDB fibers upon application of P188 and inverted triblock copolymers. Consequently, these copolymer-treated fibers showed elevated twitch peak sarcomere length shortening. Based on our observations, we posit that the interface and physical stabilization of the fragile membrane by copolymers in dystrophin-deficient skeletal muscle improves the excitation–contraction coupling to then augment sarcomeric performance [35]. A potential Ca^2+^-dependent pathway by copolymer treatment is the restoration of excitation–contraction coupling by stabilization of ryanodine receptor (RyR1) and subsequent improvement of Ca^2+^ release from the sarcoplasmic reticulum [60].

Another interesting finding of this study is the pattern of the elongated twitch sarcomere length relaxation kinetics and Ca^2+^ decay. Specifically, several of the copolymer treatments tested in this study displayed greater time to 75% baseline for sarcomere length relaxation and Ca^2+^ transient decay, as compared to BL10. We propose that this slower fiber relaxation induced by the copolymer treatment could contribute to enhanced force output via multiple twitch contraction summation [61]. Although speculative, this could further assist the force generation in dystrophin-deficient skeletal muscle.

Another potential mechanism in the restoration of contractile function by copolymers centers on a Ca^2+^-independent mechanism. Interestingly, diblock copolymers in this study showed 50% improvement in the twitch peak sarcomere length shortening in mdx FDB fibers, without significant effects on the peak Ca^2+^ transient or kinetics. These results can be discussed in the context of copolymers studied in in silico molecular dynamic models [50]. Here, the insertion of copolymers into the synthetic lipid bilayer significantly increased the lateral pressure required to rupture the bilayer, thus underlying the mechanism of membrane protection. Another study injecting copolymers in lipid monolayer models in the Langmuir trough model detected an immediate increase in surface pressure [35]. Atomic force microscopy also showed that the morphology of a lipid monolayer was altered by the copolymer insertion [62]. In computational models, copolymers modify the structure of the membrane by interacting with the polar head group in the lipid bilayer [63]. These findings suggest that copolymers localize at the membrane and do not enter into the fiber. Taken together, the localization and interaction of exogenous synthetic copolymers in the membrane of muscle fiber alter the physical features such as viscosity, stiffness, and tension of the membrane, consequently leading to improvements in the force production of the myofibers.

The modifications in the contractile function of the mdx skeletal muscle fibers induced by synthetic membrane stabilizing copolymer treatments are shown here to be combined results of both Ca^2+^-dependent and Ca^2+^-independent mechanisms. The involvement of the peak Ca^2+^ transient by three distinct copolymers suggests that the diblock copolymer utilizes a Ca^2+^-independent pathway, potentially due to its distinct structure (2 blocks, -C_4_ end group) and lower molecular weight. Considering the rapid functional modifications and localization of copolymer at the membrane of the myofibers without entering into the cells, these protective mechanisms exclude protein biosynthesis or post-translational modification as essential features of membrane stabilization by copolymers [9].

### Structure of copolymers

Block copolymers can be designed in various combinations by modifying molecular weight, the number of blocks, degree of polymerization, hydrophobicity, chemical end group, and architecture [25, 37, 40, 51, 64]. As the first study in the field to apply synthetic copolymers to the primary, live, membrane-intact, single skeletal muscle fibers, we tested three block copolymers with different structures to mdx FDB fibers. The most distinctive differences in the copolymers utilized in this study were number and orientation of blocks as well as molecular weight, while maintaining monomer identities and numbers of PEO (hydrophilic) and PPO (hydrophobic) and relative composition (~ 80% PEO), as presented in Table 1. All copolymers tested markedly improved the twitch sarcomere length amplitude in the mdx FDB fibers compared to vehicle. Interestingly, these data also showed some differences in effectiveness, as dictated by the structure–function relationship of these copolymers. One of the common features of the three copolymers used in this study is the amphiphilic architecture. The working model of membrane stabilization includes the interface of hydrophobic PPO block(s) into the membrane and stabilizing the molecule by the hydrophilic PEO block [37]. Importantly, the hydrophobicity of the copolymers via the PEO:PPO ratio strongly influences the effectiveness of stabilizing the lipid bilayer [63]. In this study, molecular weight and relative location of PPO and PPO blocks were varied factors, as their PEO weight ratios were relatively similar (P188 = 80% PEO, diblock copolymer = 77% PEO, inverted triblock copolymer = 83% PEO).

### Limitations

This study utilized isolated skeletal muscle fibers which offer minimal diffusion barrier and maximal surface area for the membrane stabilization copolymers activation. The advantage of this approach is the insight into the mechanism of membrane stabilizing copolymers on the short-lived experimental window of isolated skeletal muscle. Additional extensive study is required to further investigate these effects using animal models of DMD in vivo.

## Conclusions

This study reports rapid and robust functional restitution in the twitch sarcomere shortening capacity of primary, live, dystrophin-deficient skeletal muscle fibers by synthetic copolymer treatment. This provides evidence for the membrane stabilization function of copolymers as a potential therapeutic approach for Duchenne muscular dystrophy patients. Furthermore, data show effectiveness in the rescue-of-function by the engineering of diverse copolymer structures. Lastly, considering the rapidity of the functional restitution, data implicate both Ca^2+^-dependent and Ca^2+^-independent intracellular pathways as potential mechanisms underlying the effectiveness of membrane stabilizing copolymers. This work adds to a growing literature supporting the copolymer—muscle membrane interface as a driving element underlying improve muscle fiber performance in the setting of dystrophin deficiency.

## Supplementary Information

**Additional file 1:** Molecular characterization of diblock and inverted copolymers. **Figure S1.** Characterization of diblock (green) and inverted (red) copolymers. Application of P188 in dystrophin-replete FDB fibers from C57BL/10 control mice. **Figure S2.** Pairwise comparison of peak sarcomere length shortening during twitch contraction from dystrophin-replete FDB fibers from C57BL10 mice (3 mice, 32 fibers) in pre- vs. post-P188 treatment at 150 µM (P = 0.359). Duration of field stimulation pulse. **Figure S3.** Comparison of peak sarcomere length shortening during twitch contraction from mdx mice.

## Data Availability

All data generated or analyzed in this study are included in this published article (and its supplementary information files). The datasets generated during and/or analyzed during the current study are available from the corresponding author on reasonable request.

## Abbreviations

ANOVA: Analysis of variance
DMD: Duchenne muscular dystrophy
EDL: Extensor digitorum longus
FDB: Flexor digitorum brevis
PPO: Poly(propylene oxide)
PEO: Poly(ethylene oxide)
SERCA: Sarco/endoplasmic reticulum Ca^2+^-ATPase
SL: Sarcomere length
THF: Tetrahydrofuran
