# Supplementary material for: Rapid restitution of contractile dysfunction by synthetic copolymers in dystrophin-deficient single live skeletal muscle fibers
Source: Skelet Muscle. 2023 May 19;13:9. doi: 10.1186/s13395-023-00318-y (PMC10197332; doi:10.1186/s13395-023-00318-y)
Supplement: Supplementary file 1 — Additional file 1: Molecular characterization of diblock and inverted copolymers. Figure S1. Characterization of diblock (green) and inverted (red) copolymers. Application of P188 in dystrophin-replete FDB fibers from C57BL/10 control mice. Figure S2. Pairwise comparison of peak sarcomere length shortening during twitch contraction from dystrophin-replete FDB fibers from C57BL10 mice (3 mice, 32 fibers) in pre- vs. post-P188 treatment at 150 µM (P = 0.359). Duration of field stimulation pulse. Figure S3. Comparison of peak sarcomere length shortening during twitch contraction from mdx mice. [file 13395_2023_318_MOESM1_ESM.pdf]

Supplementary information

Molecular characterization of diblock and inverted copolymers

Polymer composition, molecular weight, and dispersity were determined by peak analysis of ^{1}H NMR spectra of polymer in deuterated chloroform (NMR; 400 MHz Bruker Avance III HD nanobay AX-400). Polymer dispersity was determined using size exclusion chromatography (SEC) calibrated with polystyrene standards and tetrahydrofuran (THF) as the solvent. Molecular weights and dispersity were confirmed by matrix assisted laser desorption/ionization time of flight mass spectrometry (MALDI-TOF; TOF 5800, Sciex) with α-cyano-4-hydroxycinnamic acid (Sigma) as a matrix.

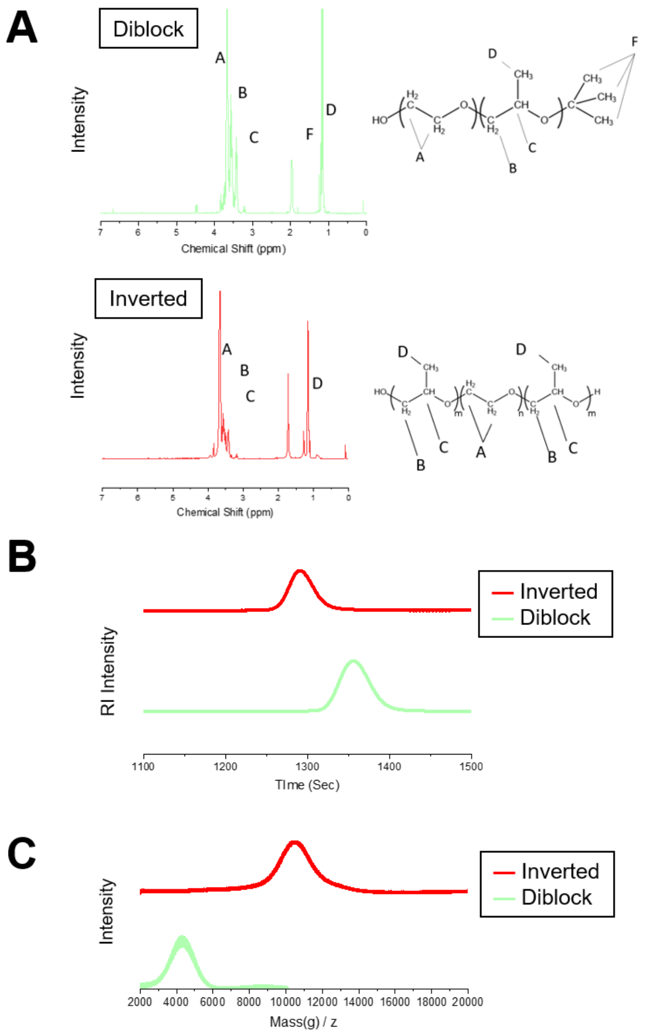

Figure S1. Characterization of diblock (green) and inverted (red) copolymers. (A) Nuclear magnetic resonance (NMR), (B) size exclusion chromatography (SEC), and (C) matrix assisted laser desorption/ionization time-of-flight (MALDI-TOF) confirm a diblock copolymer with 16 units of propylene oxide, 75 units of ethylene oxide with a dispersity of 1.06 with a molecular weight of 4,200 g/mol, and a triblock with 15 units of propylene oxide, 200 units of ethylene oxide with a dispersity of 1.05 with a molecular weight of 10,700 g/mol.

Application of P188 in dystrophin-replete FDB fibers from C57BL10 control mice

Dystrophin-replete FDB fibers from C57BL/10 mice were tested in a pairwise comparison approach as pre- vs. post-P188 treatment at 150 µM. Compared to the vehicle, P188 150 µM treatment had no statistical effect on the peak sarcomere length shortening from the dystrophin-replete FDB fibers (P = 0.359).

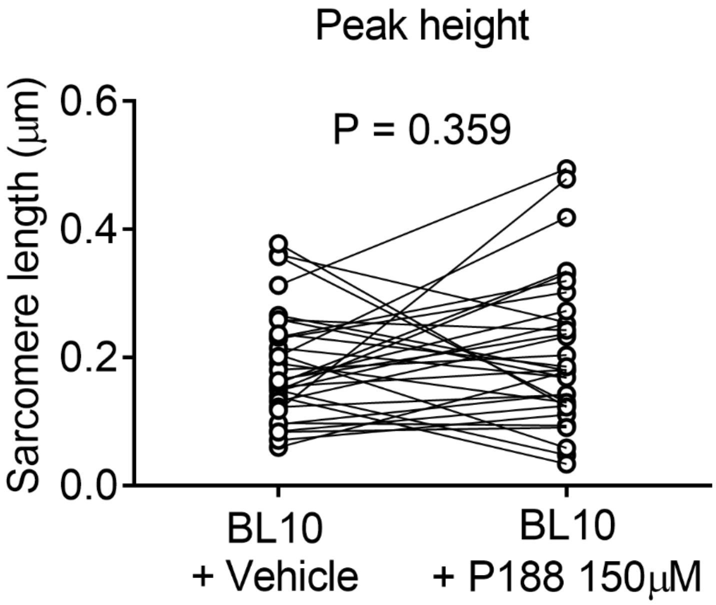

Figure S2. Pairwise comparison of peak sarcomere length shortening during twitch contraction from dystrophin-replete FDB fibers from C57BL10 mice (3 mice, 32 fibers) in pre- vs. post-P188 treatment at 150 µM (P = 0.359).

Duration of field stimulation pulse

We tested a range of field stimulation pulses to FDB fibers from mdx mice. The results showed that 8 ms, which was applied in this study, elicited numerically the greatest peak sarcomere length shortening.

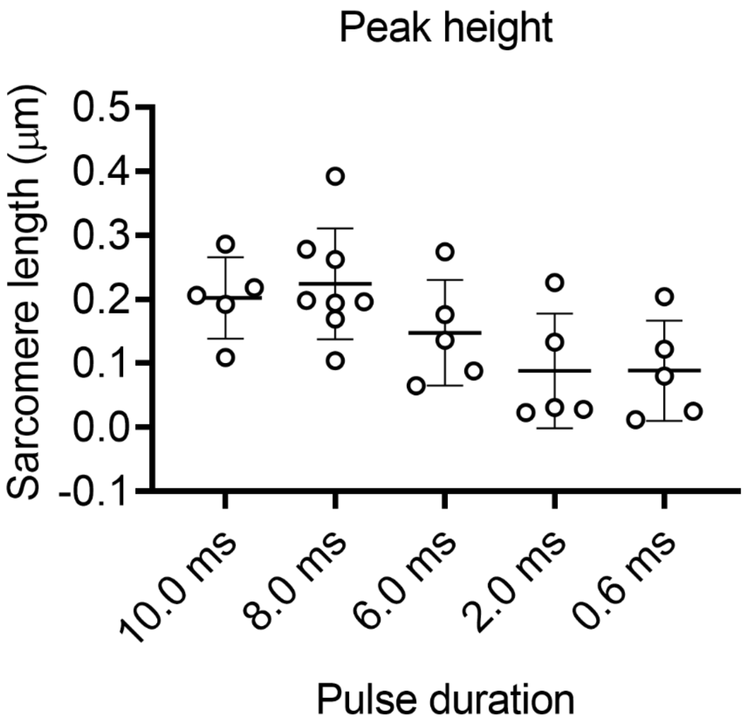

Figure S3. Comparison of peak sarcomere length shortening during twitch contraction in FDB fibers from mdx mice.
